# Expert survey on coverage and characteristics of pediatric palliative care in Europe – a focus on home care

**DOI:** 10.1186/s12904-022-01078-0

**Published:** 2022-10-17

**Authors:** Julia Wager, Larissa Alice Kubek, Maria Brenner, Sara Calmanti, Carmel Doyle, Malin Lövgren, Ulrika Kreicbergs, Leontien Kremer, Philippe Le Moine, Guillaume Robert, Meggi Schuiling-Otten, Peter Schröder-Bäck, Eduard Verhagen, Boris Zernikow

**Affiliations:** 1 PedScience Research Institute, Herdieckstraße 5b, 45711 Datteln, Germany; 2 Paediatric Palliative Care Centre, Children’s and Adolescents’ Hospital, Datteln, Germany; 3 Department of Children’s Pain Therapy and Paediatric Palliative Care, Faculty of Health, School of Medicine, Witten/Herdecke University, Witten, Germany; 4 School of Nursing, Midwifery & Health Systems, University College, Dublin, Ireland; 5 Accompagnement Et Information (CREAI) en Faveur Des Populations Vulnérables, Centre Régional d’Etudes, Bretagne, France; 6 School of Nursing and Midwifery, Trinity College, Dublin, Ireland; 7 Marie Cederschiöld University SE, Stockholm, Sweden; 8 Princess Máxima Center for Pediatric Oncology, 3584 CS Utrecht, The Netherlands; 9 Equipe Ressource Régionale de Soins Palliatifs Pédiatriques La BRISE, Bretagne, France; 10 Dutch Center of Expertise in Paediatric Palliative Care, Utrecht, The Netherlands; 11 Department of International Health, Care and Public Health Research Institute (CAPHRI), Maastricht University, Maastricht, The Netherlands; 12 Department of Pediatrics, University Medical Center Groningen, University of Groningen, Groningen, The Netherlands

**Keywords:** Palliative care, Pediatrics, Health care quality, access, and evaluation, Home care services, Europe

## Abstract

**Background:**

For children with life-limiting conditions home care is a key component of pediatric palliative care. However, poor information is available on service coverage and in particular on country-specific pediatric palliative home care characteristics. The aim of the study was therefore to describe the association between pediatric palliative care coverage and national activities and obtain detailed information on the pediatric palliative home care structure in different European countries.

**Methods:**

Online survey with in-country experts from *N* = 33 European countries.

**Results:**

Pediatric palliative home care (65.6%) represented the most pediatric palliative care units (15.6%) and the least common services. National documents constituted the most widespread national pediatric palliative care activity (59.4%) and were associated with available services. Pediatric palliative home care could be mostly accessed as a service free of charge to families (95.2%) from the time of a child's diagnosis (85.7%). In most countries, oncological and non-oncological patients were cared for in pediatric palliative home care. Only a minority of home care teams covered home-ventilated children. Pediatric palliative home care usually comprised medical care (81.0%), care coordination (71.4%), nursing care (75.0%) and social support (57.1%). Most countries had at least two professional groups working in home care teams (81.0%), mostly physicians and nurses. In many countries, pediatric palliative home care was not available in all regions and did not offer a 24 h-outreach service.

**Conclusions:**

Pediatric palliative care provision in Europe is heterogeneous. Further work on country-specific structures is needed.

**Supplementary Information:**

The online version contains supplementary material available at 10.1186/s12904-022-01078-0.

## Background

Although no figures are available for each single country—based on figures from England and Italy—20–66 per 10.000 children in Europe are affected by life-limiting conditions requiring pediatric palliative care [1, 2]. Pediatric palliative care refers to an active and all-encompassing care approach that in many cases starts upon diagnosing a child's life-limiting illness and often supports families for many years, while not curing but rather achieving the utmost level of comfort representing the overarching goal [3–7].

Today, pediatric palliative care is no longer limited to inpatient treatment, but is primarily provided in the home environment [8–10]. Due to the young patients' frequent sector switches and the high demand for professional expertise from various disciplines, comprehensive, closely networked pediatric palliative care is indispensable [11–13]. As an important framework condition for this, pediatric palliative care should be integrated into national legislation and strategies [14].

For some years now, endeavors have been underway to systematically assess the current national and global provision of pediatric palliative care. In 2011, based on a classification initially applied to adult palliative care [15], a study for the first time systematically addressed the global pediatric palliative care development status by assigning 115 individual countries to one of four levels of pediatric palliative care (no known pediatrics hospice-palliative care activity, capacity building activity, localized provision, integration with mainstream providers [16];). In 2017, based on an expanded version of this classification, the global pediatric palliative care development was again assessed according to judgments by in-country experts and not on the basis of existing data. Of the 113 countries included in this study, only 7 countries achieved the highest level representing the most advanced level of integrated pediatric palliative care. Twenty-one countries reported no pediatric palliative care activity at all [17]. Although these studies provided important general findings, the authors themselves discussed that the defined levels may not optimally reflect the actual realities due to their rigidity [16]. Detailed information on pediatric palliative care characteristics was not assessed [16, 17].

In 2019, the European Association for Palliative Care (EAPC) released a first-ever structured and standardized overview of palliative care development for 54 individual European countries [7, 18]. In this EAPC Atlas, alongside statements on palliative care for adult patients, consideration was also given to the pediatric palliative care context.

In a study akin to the EAPC Atlas presenting data from 2018–2019, it was shown that in 48 of 51 countries a total of 680 pediatric palliative care services were identified; provided in hospitals, hospices and the home environment. 37% to 42% of countries designated specific national pediatric palliative care activities (e.g., norms and standards, associations). In 20 countries (39%), the presence of pediatric palliative care specialists were reported [19]. Overall, this information indicates that there seems to be a noticeable imbalance between countries, with some countries not offering any pediatric palliative care services [7, 18]. To further monitor pediatric palliative care development in Europe, in-depth information on its current status needs to be collated. Of particular interest is the pediatric palliative home care status. Evidence shows that the support of pediatric palliative home care teams can reduce parents’ burden, strengthen their caregiving skills, and optimize the situation of young palliative care patients [20, 21]. Ideally, these home care teams should be a) organized multi-professionally and as a significant premise take into account the individuality of each child and family with their values, desires and needs, b) be available around the clock have sufficient expertise to meet the physical, psychological, emotional, spiritual and social needs of children and families and c) to offer them comprehensive support and supervision [22]. In addition, for many families, the home environment is the desirable place of death for the affected child, a wish that can more likely be fulfilled through the involvement of pediatric home care teams [21, 23].

The aim of this study was to obtain detailed information on pediatric palliative care coverage and national activities in European countries based on sound expert knowledge. A special focus was placed on pediatric palliative home care.

## Materials and methods

### Survey instrument

For the online questionnaire, potential items were identified through a literature search and additionally formulated by professionals of a pediatric palliative care facility in Germany. They had many years of in-depth knowledge in the care of pediatric palliative patients and in the current research and literature on pediatric palliative care. Therefore, the supplemented items resulted from identified knowledge gaps that were collectively deemed relevant.

These items were then presented to pediatric palliative care professionals in different European countries. Their feedback was discussed by the team putting together the initial questionnaire, and adaptations to the questionnaire were made. The adapted questionnaire was then circulated within a smaller international group via e-mail until all participants reached agreement regarding relevance, applicability and comprehensibility of the adaptations. The draft questionnaire was translated into an online version using the QuestionPro platform (Berlin, https://www.questionpro.de) and sent for a pre-test to 10 test respondents with pediatric palliative care expertise. Based on their feedback, the questionnaire was revised and finalized. The final questionnaire structure first enquired about the existence of different national pediatric palliative care activities and services. If the availability of pediatric palliative home care teams was indicated, detailed questions followed (please see Supplemental Material 1 and 2).

### Participants

For answering the final questionnaire, pediatric palliative care experts were sought in the total of 50 EU member states, candidate countries and further countries in Europe according to the European Commission's definition (excluding the Russian Federation, the Vatican City State; [24]) between March 2020 and February 2021. An internet search in e.g., the literature search engine PubMed, Google, career portals (e.g., Xing, LinkedIn) and social/research networks (e.g., ResearchGate, Facebook) was conducted targeting people that had experience with pediatric palliative care. Additional contact people were identified through personal networks and national expert associations.

### Data collection

By means of a standardized e-mail, these individuals were informed about the study background and asked whether they would consider themselves sufficiently qualified for providing reliable information on pediatric palliative care in their country. If this was not the case, they were invited to name a potential informant. Those who confirmed having the necessary qualifications and wished to complete the questionnaire were sent it together with the electronic study information and consent form.

Ethics approval for the study was obtained from the clinical ethics committee of the Children’s and Adolescents’ Hospital Datteln, Germany (approval code: 2022/04/20/BZ).

### Data analysis

The questionnaire data were extracted and converted into the SPSS file format (IBM, version 27). All analyses were performed at country-level using descriptive statistics. Point biserial correlations were calculated to determine possible relationships between the presence and number of national pediatric palliative care activities and services. Because of expected cell frequency less than 5, associations between the presence of national pediatric palliative care activities and of pediatric palliative care services were determined using the Fisher-Freeman-Halton exact test. Graphical geographical depictions were generated via the free online tool MapChart (https://mapchart.net/).

## Results

### General characteristics

Experts from *N* = 38 countries responded to the online questionnaire. Multiple datasets were submitted from some countries. In this case, whichever questionnaire provided the most comprehensive data was kept (exclusion of *n* = 5, 13.15%, questionnaires; final dataset: *N* = 33 countries).

Respondents had median 14 years (M = 13.5; SD = 9.8, range = 0–40 years) of first-hand pediatric palliative care experience with the majority being physicians (*n* = 21, 65.6%; nurses: *n* = 2, 6.3%; researchers: *n* = 2, 6.3%; other: *n* = 7, 21.9%; missing information: *n* = 1). Experts were chosen based on their expertise in PPC (Table 1).

**Table 1:** Involvement in PPC as indicated by the experts surveyed (*N* = 33)

Involvement^a^	*n* (%)
Actively providing care	19 (57.6)
Administrative services	9 (27.3)
Active membership in pediatric palliative care networks/associations	15 (45.5)
Passive membership in pediatric palliative care networks/associations	1 (3.0)
Other	15 (45.5)
Academic / Education	10 (30.3)
Advocacy	5(15.2)

^a^Multiple entries possible

**Fig. 1:**
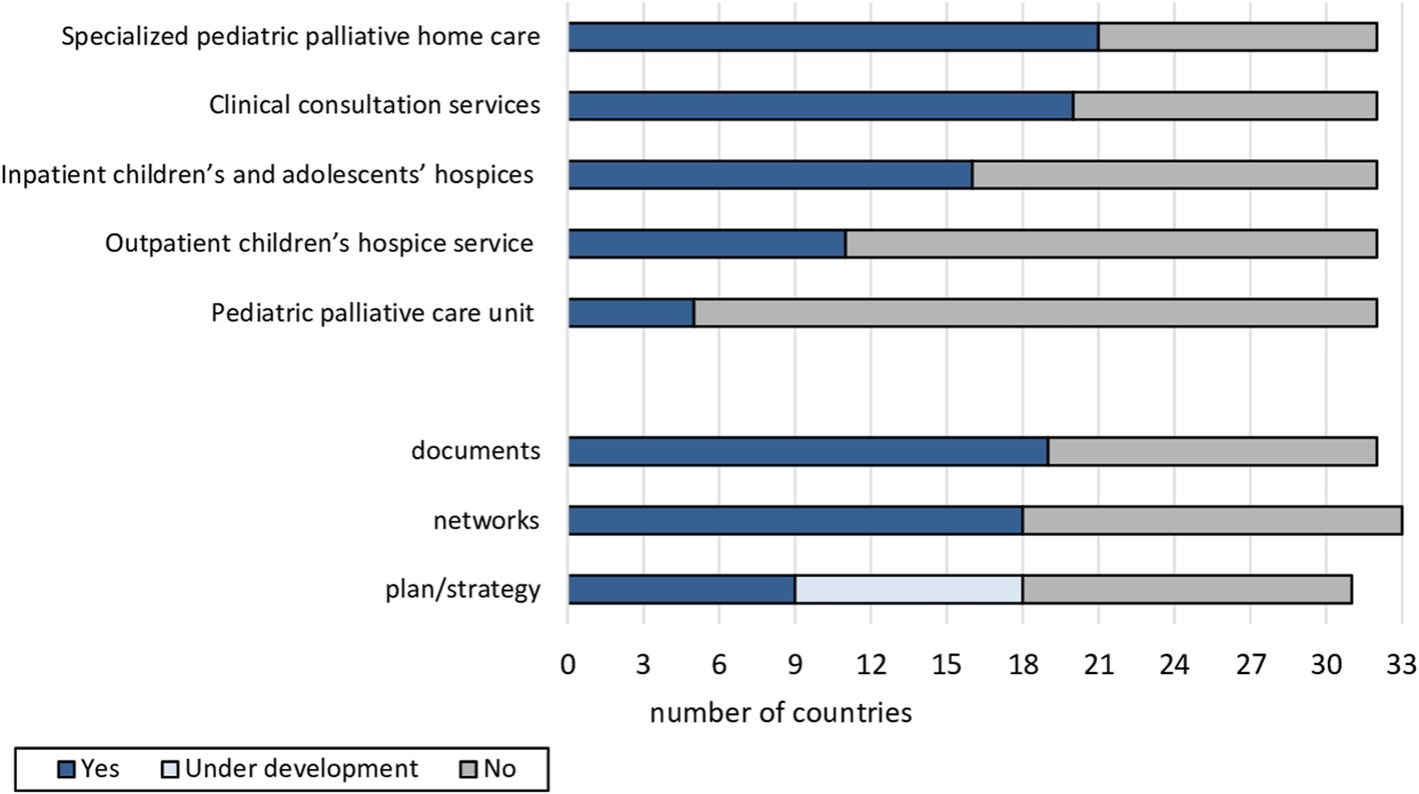
Presence of pediatric palliative care services and national pediatric palliative care activities in the *N* = 33 European countries surveyed

Across all countries, pediatric palliative home care represented the most and pediatric palliative care units the least pervasive (*n* = 21, 65.6% versus *n* = 5, 15.6% respectively) service. The existence of national documents and professional networks was confirmed by more than half of the countries surveyed (documents: *n* = 19, 59.4%; networks: *n* = 18, 54.5%; Fig. 1). A national plan or strategy existed in *n* = 9 (27.3%) countries and was likewise under development in *n* = 9 (27.3%) countries.

### Country-specific characteristics

No pediatric palliative care service or national activity could be identified in Albania and Malta. In Armenia, solely a national plan/strategy was under development. Spain alone was able to affirm the existence of all inquired activities and services. The largest number of pediatric palliative home care (*n* = 66) was reported in Poland, of clinical consultation services in Spain (*n* = 30), of pediatric palliative care units in Turkey (*n* = 7), of inpatient children’s and adolescents’ hospices in the United Kingdom (*n* = 54) and of outpatient children’s hospice services in Germany (*n* = 165; Table 2).

**Table 2:**
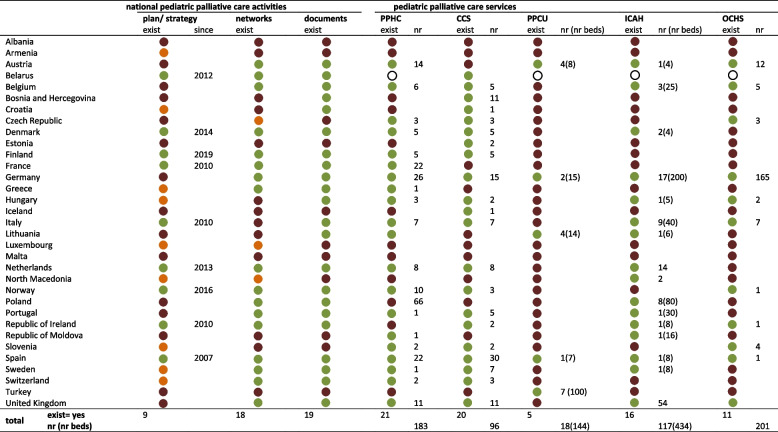
Availability of national pediatric palliative care activities and pediatric palliative care services per country^a^

existent  non-existent  under development  not stated

^a^*Exist* Existence of, *nr* Number of, *PPHC* Pediatric palliative home care, *CCS* Clinical consultation services, *PPCU* Pediatric palliative care unit, *ICAH* Inpatient children’s and adolescents’ hospices, *OCHS* Outpatient children’s hospice service

### Interrelations between national pediatric palliative care activities and services

A significant negative correlation was identified between the number of pediatric palliative care unit beds and the existence of a country’s national pediatric palliative care documents (*r* = -0.99, *p* < 0.01). No other significant associations were found between national PPC activities’ existence and the number of established PPC services.

Among the national activities, a significant association was solely evident between the existence of a national plan/ strategy and national networks (Fisher-Freeman-Halton exact test, *p* < 0.05, Cramer’s *V*: 0.49).

## Pediatric palliative home care

### Patients in SPPHC

**Fig. 2:**
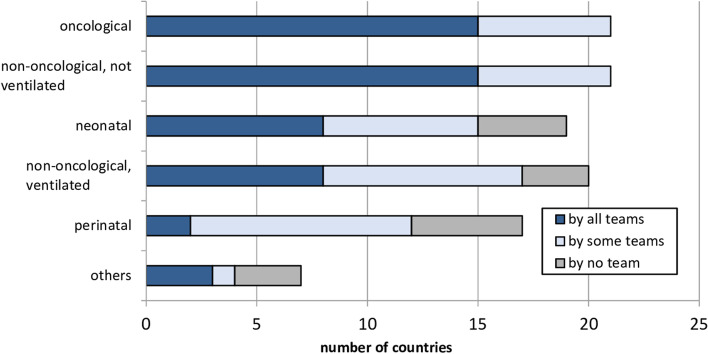
Patients cared for by pediatric palliative home care teams

In *n* = 18 (85.7%) of the *n* = 21 countries generally confirming the existence of pediatric palliative home care, patients could take advantage of the service from the time their illness was diagnosed (only during end-of-life period: *n* = 3 countries, 14.3%). The care of oncological and of non-oncological / non-ventilated patients was generally confirmed by all countries, while other patient groups were not cared for by any home care team in some countries (Fig. 2). Most respondents cited an age of 18 years (*n* = 14, 66.7%) as patients’ upper age limit for eligibility for pediatric palliative home care (16 years: *n* = 1, 4.8%; 19 years: *n* = 2, 9.5%, 21 years: *n* = 3, 14.3%; 23 years: *n* = 1, 4.8%).

### Pediatric palliative home care access

In the majority of countries, pediatric palliative home care was neither limited in time (e.g., to 4 weeks per year; not limited: *n* = 19, 90.5%; limited: *n* = 2, 9.5%) nor associated with private costs (free of charge: *n* = 20, 95.2%; private charges: *n* = 1, 4.8%). The service was mainly funded by government (*n* = 9 countries, 42.9%), followed by health insurances (*n* = 7 countries, 33.3%), donations (*n* = 2 countries, 9.5%) and other sources (*n* = 3 countries, 14.3%). Access was given by referral from a physician in most countries (*n* = 15, 71.4%; privately arranged: *n* = 4 countries, 19%; and other: *n* = 2 countries, 9.5%). The home care team location was mainly exclusively (*n* = 8, 38.1%) or mostly (*n* = 3; 14.3%) hospital-based, in *n* = 6 (28.6%) countries mostly community-based and in *n* = 4 countries (19.0%) exclusively community-based.

In *n* = 12 countries (57.1%), home care teams were only available in one or a few regions, and in *n* = 5 countries (23.8%) in most regions.

### Pediatric palliative home care team composition and services offered

**Fig. 3:**
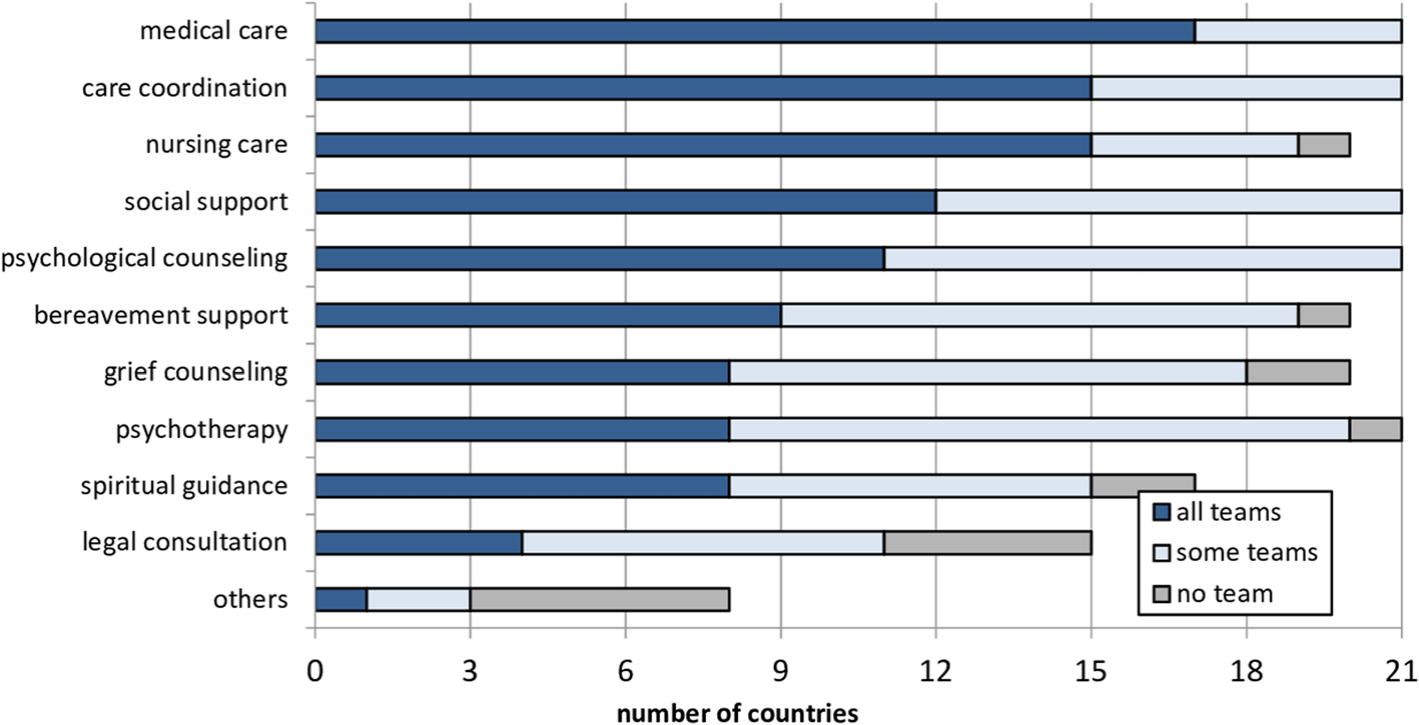
Pediatric palliative home care teams’ service scope

Medical care (*n* = 17, 81.0%), nursing care (*n* = 15, 71.4%) and care coordination (*n* = 15, 71.4%) were most frequently named as being offered by all pediatric palliative home care teams in a country (Fig. 3).

In *n* = 17 (81.1%) countries with pediatric palliative home care, at least two professions were engaged in all national teams (indicating multi-professionalism, please see Supplemental Material 3). In all multi-professional teams, physicians and nurses worked together. The greatest number of different professional groups involved in all home care teams was reported by the Czech Republic. In the Republic of Moldova, no professional group was consistently represented in all pediatric palliative home care offerings; the professions varied widely**.** In Spain, Belgium, and Austria only one professional group was consistently represented in all pediatric palliative home care offerings in each country.

**Fig. 4:**
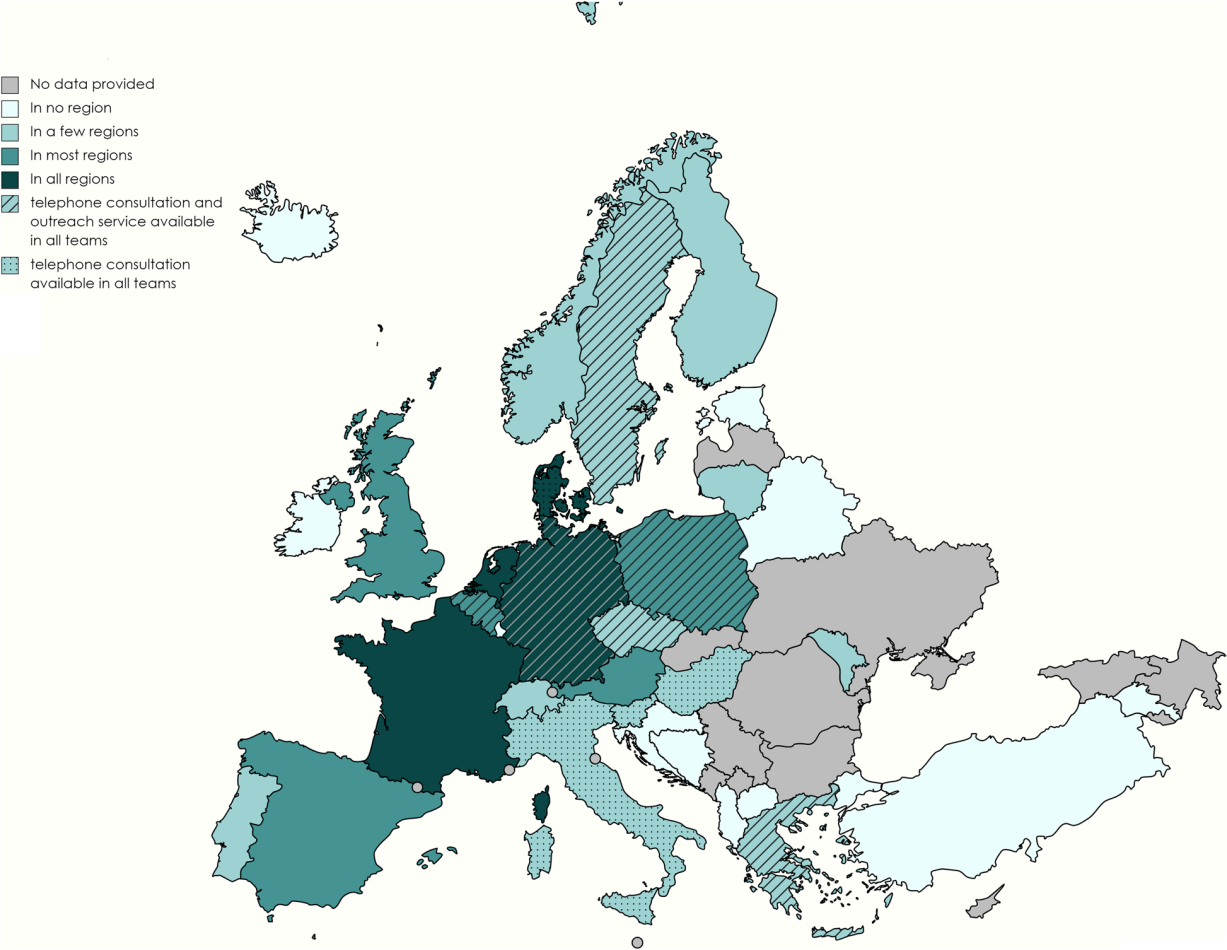
Regional availability of pediatric home care in *n* = 17 European countries and availability of 24/7 telephone consultation and outreach service in all home care teams

Solely in the Netherlands, France, Denmark and Germany, pediatric palliative home care (*n* = 4, 19.0%) was confirmed as being available in all regions. For these countries, multi-professionalism across all national home care teams could also be noted (median = 4, range = 2–6 different professional groups; Fig. 3). 24/7 telephone consultation was offered in *n* = 10 (47.6%) and 24/7 outreach service in *n* = 6 (28.6%) countries by all teams (Table 3 and Fig. 4).

**Table 3:**
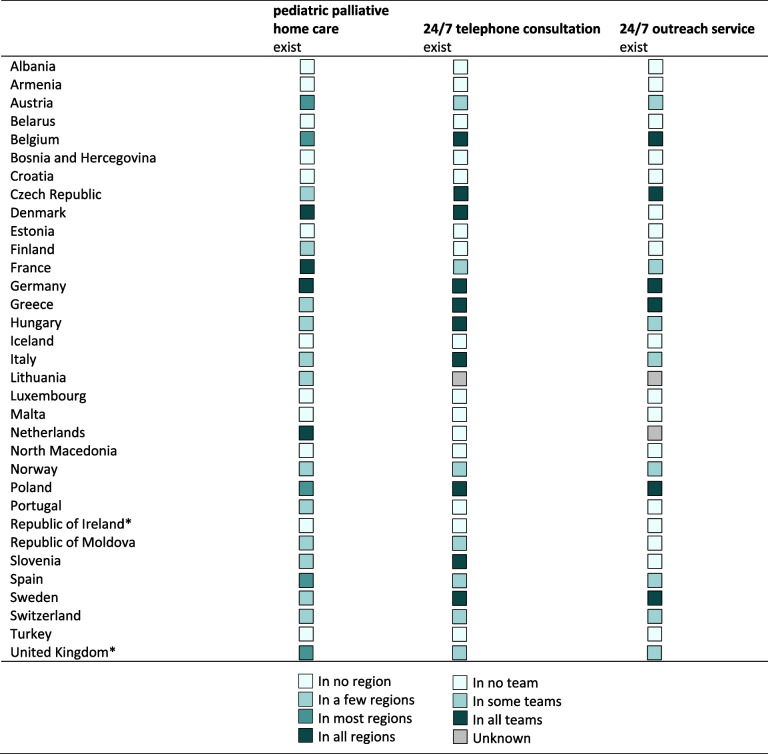
Regional availability of pediatric palliative home care and availability of 24/7 telephone consultation and outreach service in countries' existing home care teams

## Discussion

### Main findings

The present study aimed to provide detailed information on the provision situation and concrete services of pediatric palliative care in Europe. Overall, pediatric palliative home care was identified as the most widespread pediatric palliative care service, which is consistent with existing literature [18]. At the country level, the results on existing pediatric palliative care services differed partly from the “EAPC Atlas of Palliative Care in Europe 2019” data [18] and the data reported by Arias-Casais et al. [19]. As a possible cause for discrepancies like these, the chosen descriptions and naming of the queried services may be considered. For example, for pediatric palliative care units the study did not inquire about pediatric palliative care teams in acute hospitals, but rather explicitly asked about units solely admitting pediatric palliative patients. Although both wordings ask for similar information at their core it is nevertheless conceivable that the key experts evaluated and listed different services than in other surveys [17, 18, 25, 26].

The existence of national plans or strategies was inquired separately from the existence of official pediatric palliative care documents. Even though both aspects can theoretically coincide, since national plans and strategies may also be delineated in official documents, the reason for this was that there could theoretically also be disparities between the two aspects. The study results support this assumption showing many countries had pediatric palliative care documents, but no national plans or strategies available; nor could any correlations between the two aspects be identified. Another reason for the discrepancy between our findings and those of the EAPC Atlas could be divergent professional backgrounds and information of the informants that we interviewed. For example, if an informant is a healthcare professional with global expertise and overview of the national healthcare system, but is not an active healthcare worker himself (in pediatric palliative care), she or he may have different information than a person who is "close to the action" and actively follows national developments in pediatric palliative care. To avoid any discrepancies, fixed criteria should be developed and evaluated in the future regarding which individuals should be considered as informants for studies such as the present one. Linkages between the national pediatric palliative care activities and individual pediatric palliative care services were identified. Possible indicators of the extent to which pediatric palliative care is integrated into a country's health care system have been pointed out and are reflected in the pediatric palliative care services we surveyed (for example, number of pediatric palliative care consultants [27]). Our results may therefore suggest that the pediatric palliative care services surveyed may indeed be good indicators for examining the current integration of pediatric palliative care in a national healthcare system and may already reflect certain developing tendencies of countries [27].

We identified a negative association between the number of pediatric palliative care unit beds and whether a country had national pediatric palliative care documents. This result may also reflect a country's efforts to establish stronger pediatric palliative care and improve the state of current care. If only few practical implementations of pediatric palliative care beds exist, then national documents can be used to better expand these structures in the future. Ultimately, however, we cannot draw any causal conclusions in this study. This and other associations should be explored further in follow-up studies. Across all countries, we were generally able to show a broad range of services offered by pediatric palliative home care teams with none of the queried services not being offered in any country. Further, in all but three countries, the in-country experts confirmed the existence and availability of pediatric palliative home care at no cost to families, which is encouraging given that young patients and their families primarily desire care in the home setting [8]. This underlines the important role national policies can play.

In all but four countries, pediatric palliative home care teams were organized multi-professionally and thus aligned with the core rationale of pediatric palliative care [2, 3]. Medical and nursing staff was present in all multi-professional home care teams in all countries and thus can be considered a core component or basis of the pediatric palliative home care team. The composition of the teams showed a rather heterogeneous picture across all countries.

Some results appear surprising. For example, some of the apparently straightforward home care services that one would expect in pediatric palliative care such as medical or nursing care and care coordination were actually delivered in a number of countries by only a few teams or were not at all delivered by pediatric palliative home care teams. To obtain more information on the quality and scope of services of individual home care teams in a country and across Europe, specific (questionnaire) measures should be used in the next step. One such instrument may be the recently developed “EXPERIENCE@Home” which assesses families’ experiences with pediatric palliative home care, e.g., regarding physical aspects and continuity of care [28]. Especially for premature babies or critically ill newborns and for ventilated patients, care by pediatric palliative home care teams is not guaranteed in all European countries. Perhaps, teams do not care for perinatal and neonatal patients in some countries because they are not integral part of the care network. In the long term, the networking of pediatric palliative care services and other disciplines is crucial for providing families with the best possible support, especially regarding the care of their child at home [9, 12, 20, 29].

### Limitations

A major limitation of the study concerns its data basis. Even if explicitly pediatric palliative care experts were surveyed in the respective countries, this appraisal was ultimately based on single respondents’ subjective judgments. The discrepancy between other existing studies and the results of this study may be due to the fact that the people consulted possessed different information and perspectives which cannot be conclusively objectified [18, 19]. An interesting approach to systematically combine different sources of information is the so-called “community needs assessment (CNA)”, through which the resources and needs of a particular area can be thoroughly identified and information provided for future programming and policy [8]. A CNA has already been applied to the American state of Georgia to gather the respective pediatric palliative care resources as comprehensively as possible [8, 30]. For the present study, a CNA was not chosen because it was first necessary to create an overview of whether and what kind of access to informants is actually possible in the European countries. In principle, the (subjective) questioning of experts can never be completely eliminated and represents a significant information base; however, it should be equipped with further information accesses in order to allow a more comprehensive picture of the pediatric palliative care situation. Our study with the corresponding methodology can represent an important first step in this direction. Even though the definitions of the pediatric palliative care services we surveyed were developed in collaboration with colleagues across Europe, it remains conceivable that we did not fully capture the pediatric palliative care situation in some countries. By incorporating different types of information sources to find out how pediatric palliative care is defined in a given country and where it actually takes place in the healthcare system should be addressed in future projects.

The present study only applies to select European countries. On the one hand, our data do not allow us to describe the overall European pediatric palliative care status, and on the other hand, more complex statistical analyses were not possible due to the relatively small number of cases. For example, it would be interesting to see whether model-like links and causalities between national pediatric palliative care activities and services can be identified. Further studies involving additional countries are required to facilitate detailed statements on the global pediatric palliative care situation.

## Conclusions

This study provides important insights into the current structure of pediatric palliative home care services in 33 European countries. Specific information, particularly on pediatric palliative home care, was obtained to gain a deeper understanding of the pediatric palliative care situation.

## Supplementary Information

**Additional file 1: Supplemental Material 1.** Online questionnaire on PPC structures in Europe used for the study purpose.

**Additional file 2: Supplemental Material 2.** Topic blocks and definitions within the utilized pediatric palliative care questionnaire.

**Additional file 3: Supplemental Material 3.** Specialized pediatric palliative home care team composition.

## Data Availability

The datasets generated and analyzed during the current study are not publicly available for reasons of privacy. They are however available (fully anonymized) from the corresponding author on reasonable request.

## Abbreviations

CAN: Community needs assessment
EAPC: European Association for Palliative Care
EU: European Union
PPC: Pediatric Palliative Care
