# Supplementary material for: Expert survey on coverage and characteristics of pediatric palliative care in Europe – a focus on home care
Source: BMC Palliat Care. 2022 Oct 17;21:185. doi: 10.1186/s12904-022-01078-0 (PMC9575204; doi:10.1186/s12904-022-01078-0)
Supplement: Supplementary file 1 — Additional file 1: Supplemental Material 1. Online questionnaire on PPC structures in Europe used for the study purpose. [file 12904_2022_1078_MOESM1_ESM.pdf]

Supplemental Material 1. Online questionnaire on PPC structures in Europe used for the study purpose.

Which country are you answering the questionnaire for?

In the following, we will ask you about general information regarding national regulations for PPC in your country.

Do you have a national plan or strategy for PPC in your country?

1. Yes
2. No
3. Under developement
4. Other relevant plans or strategy
5. I do not know

1. When was the national plan or strategy published? Please indicate the respective year, if possible. In case you cannot make an educated guess, please enter "000"

2. How long has the plan or strategy been in effect? Please report the approximate number of years. In case you cannot make an educated guess, please enter "000"

What are the main objectives for PPC included in the plan or strategy?

4. Does an implementation plan for the national plan or strategy exist?

1. Yes
2. No
3. I do not know

Do you have national networks, associations or taskforces specifically designated for PPC? (A national network of PPC is formed by people interested in the advancement of PPC provision in a specific country and must be a registered association)

1. Yes
2. No
3. I do not know
4. Other

The network’s name in your national language:

The network’s name in English:

The network‘s internet address:

At this point, participants could indicate additional networks.

Do you have regional networks, associations or taskforces specifically designated for PPC?

1. Yes
2. No
3. I do not know

The network's name in your national language:

The network's name in English:

The network's internet address:

At this point, participants could indicate additional regional networks.

Do any published national documents regarding standards and norms for the provision of PPC exist in your country? Official documents specify requirements that PPC services should adhere to. They also demonstrate guidelines for the development, equipment and configuration of services required for adequate structural quality.

1. Yes
2. No
3. Under development
4. I do not know
5. Other

In the following, we will ask you about specific information regarding specialized PPC services available in your country. We will ask you about: Specialized Pediatric Palliative Home Care (SPHHC), Clinical Consultation Services, Pediatric Palliative Care Units, Inpatient children's and adolescents' hospices, Outpatient children's hospice Services.

Are Specialized Pediatric Palliative Home Care (SPHHC) Teams available in your country? The main objective of SPHHC teams is to provide and/or coordinate palliative care for children (0-18 years old) with life-threatening or life-limiting conditions at home. The team is managed by a professional and furthermore almost exclusively consists of professionals, though it may be supported by volunteers. Note: SPPHC may be offered by the clinical consultation service. If this applies to your country, please select "yes"; on this page and later on also provide information on clinical consultation services.

1. Yes, available in my country
2. No, not available in my country

Approximate number of SPPHC teams in your country: This information is very important for us. If you do not know the exact number of teams, please try to make an educated guess. Only in case you absolutely do not know, enter "000"

Are SPPHC teams and clinical consultation services unified in one team in your country?

1. Yes, each team functions as a SPPHC team and also provides clinical consultation services
2. Not always, combined and separate teams exist
3. No, the teams are never combined but are always separate

At this point, participants could indicate additional specialized pediatric palliative home care teams.

Where do SPPHC teams exist in your country?

1. Available in all regions
2. Availabe in most regions
3. Available only in a few regions
4. Other

How are the SPPHC teams mainly financed / funded?

1. Government funding
2. Health insurances
3. Donations

4. Other

Is there additional funding for the SPPHC teams?

1. Government funding
2. Health insurances
3. Donations
4. Other

Where are the SPPHC teams based?

1. Exclusively hospital-based
2. Mostly hospital-based
3. Exclusively community-based
4. Mostly community-based

Which professions are part of the SPPHC teams?

	Part of all teams	Part of some teams	Part of no team	I do not know
Physicans	❑	❑	❑	❑
Nurses	❑	❑	❑	❑
Psychologists	❑	❑	❑	❑
Social workers	❑	❑	❑	❑
Volunteers	❑	❑	❑	❑
Spiritual Support (e.g., chaplain, rabbi)	❑	❑	❑	❑
Grief Counselor	❑	❑	❑	❑
Others	❑	❑	❑	❑

Who is mainly responsible for the management of the respective SPPHC teams?

	All teams	Some teams	No team	I do not know
Physicans	❑	❑	❑	❑
Nurses	❑	❑	❑	❑
Psychologists	❑	❑	❑	❑
Social workers	❑	❑	❑	❑
Spiritual support (e.g., chaplain,rabbi)	❑	❑	❑	❑
Others	❑	❑	❑	❑

How do the SPPHC teams support the families?

	All teams	Some teams	No team	I do not know
Care Coordination	❑	❑	❑	❑
Medical Care	❑	❑	❑	❑
Nursing Care	❑	❑	❑	❑
Social Support	❑	❑	❑	❑
Psychological counseling	❑	❑	❑	❑
Psychotherapy (support for siblings/families)	❑	❑	❑	❑
Legal consultation	❑	❑	❑	❑
Grief counseling	❑	❑	❑	❑
Bereavement support	❑	❑	❑	❑
Spiritual guidance/support (e.g., chaplain, rabbi)	❑	❑	❑	❑
24h/7d telephone consultation	❑	❑	❑	❑
24h/7d outreach service	❑	❑	❑	❑
Others	❑	❑	❑	❑

Which patients are treated by the SPPHC teams?

	By all teams	By some teams	By no team	I do not know
Perinatal	❑	❑	❑	❑
Neonatal	❑	❑	❑	❑
Oncological patients, solid tumors	❑	❑	❑	❑
Oncological patients, leukemia	❑	❑	❑	❑
Non-oncological patients (not ventilated)	❑	❑	❑	❑
Non-oncological patients (ventilated)	❑	❑	❑	❑
Other	❑	❑	❑	❑

When may the services of the SPPHC teams be used?

1. From the diagnosis of a life-limiting/life-threatening diagnosis onward, including the end-of-life period
2. Only during the end-of-life period
3. Other

How is the access to the SPPHC teams regulated?

1. Access only via referral
2. Services may be privately arranged
3. Other

What is the regular upper age restriction for the utilization of SPPHC teams?

Do you have further comments about the regular age restriction?

Is the utilization of SPPHC teams limited in time? (e.g., 4 weeks per year)

1. Yes, utilization is limited in time
2. No, utilization is not limited in time

Is the utilization of SPPHC teams free of charge for the families?

1. Yes
2. No

Do you have any further information on SPPHC teams in your country for us that we should consider when assessing the state of PPC in the EU?

Are specialized pediatric palliative care clinical consultation services available in your country? Specialized pediatric palliative care clinical consultation services work inside the hospital and support families and other physicians/nurses when children and their families have palliative care needs, irrespective of their diagnosis.

1. Yes, available in my country
2. No, not available in my country

Approximate number of specialized clinical consultation services in your country. This information is very important for us. If you do not know the exact number of teams, please try to make an educated guess. Only in case you absolutely do not know, enter "000".

Where are the specialized clinical consultation services available in your country?

1. Available in all hospitals
2. Available in most hospitals
3. Available only in a few hospitals
4. Other

How are the specialized clinical consultation services mainly financed / funded?

1. Government funding
2. Health insurances
3. Donations
4. Other

Is there additonal funding for the specialized clinical consultation services?

1. No additional funding
2. Government funding
3. Health insurances
4. Donations
5. Other

Which professions are part of the specialized clinical consultation services?

	For all teams	For some teams	For no teams	I do not know
Physicians	❑	❑	❑	❑
Nurses	❑	❑	❑	❑
Psychologists	❑	❑	❑	❑
Social Workers	❑	❑	❑	❑
Volunteers	❑	❑	❑	❑
Spiritual Support (e.g. chaplain, rabbi)	❑	❑	❑	❑
Grief Counselor	❑	❑	❑	❑
Others	❑	❑	❑	❑

How do the specialized clinical consultation services support the families?

	All teams	Some teams	No team	I do not know
Care coordination	❑	❑	❑	❑
Medical care	❑	❑	❑	❑
Nursing care	❑	❑	❑	❑
Social Support	❑	❑	❑	❑
Psychological counseling	❑	❑	❑	❑
Psychotherapy support for siblings/families	❑	❑	❑	❑
Legal consultation	❑	❑	❑	❑
Grief counseling	❑	❑	❑	❑
Spiritual guidance/support (e.g., chaplain, rabbi)	❑	❑	❑	❑
Bereavement support	❑	❑	❑	❑
24h/7d telephone consultation	❑	❑	❑	❑
24h/7d outreach services	❑	❑	❑	❑
Other	❑	❑	❑	❑

Which patients are cared for by the specialized clinical consultation services?

	By all teams	By some teams	By no team	I do not know
Perinatal	❑	❑	❑	❑
Neonatal	❑	❑	❑	❑
Oncological patients, solid tumors	❑	❑	❑	❑
Oncological patients, leukemia	❑	❑	❑	❑
Non-oncological patients (not ventilated)	❑	❑	❑	❑

Non-oncological patients (ventilated)	❑	❑	❑	❑
Other	❑	❑	❑	❑

When may the services of the specialized clinical consultation services be used?

1. From the diagnosis of a life-limiting/life-threatening diagnosis onward, including the end-of-life period
2. Only during the end-of-life period
3. Other

Is the specialized clinical consultation service a fixed component of the care children with life-limiting conditions/life-threatening conditions and their families receive in the hospital?

1. Yes
2. No
3. Other

Do any age restrictions regarding the affected child exist for the utilization of specialized clinical consultation services?

1. Yes
2. No

Is the utilization of specialized clinical consultation services free of charge for the families?

1. Yes
2. No

Do you have any further information on the specialized clinical consultation services in your country for us that we should consider when assessing the state of PPC in the EU?

Are Pediatric Palliative Care (PPC) units available in your country? A PPC unit is a hospital unit that exclusively admits PPC patients. PPC units do not provide respite care. Pediatric beds on palliative care units for adults are not considered a PPC unit.

1. Yes, available in my country
2. No, not available in my country

Approximate number of PPC units in your country. This information is very important for us. If you do not know the exact number of teams, please try to make an educated guess. Only in case you absolutely do not know, enter "000".

Approximate total number of beds of all PPC units in your country. This information is very important for us. If you do not know the exact number of teams, please try to make an educated guess, a rough information is ok. Only in case you absolutely do not know, enter "000".

Please provide the internet address of the PPC unit:

At this point, participants could indicate the internet address of additional pediatric palliative care units.

Where do PPC units exist in your country?

1. Available in all regions
2. Available in most regions
3. Available only in a few regions
4. Other

How are the PPC units mainly financed / funded?

1. Government funding
2. Health insurances
3. Donations
4. Other

Is there additional funding for the PPC units?

1. Government funding
2. Health insurances
3. Donations
4. Other

Which professions, besides physicians and nurses, work on the PPC units?

	On all units	On some units	On no unit	I do not know
Psychologists	❑	❑	❑	❑
Social workers	❑	❑	❑	❑
Volunteers	❑	❑	❑	❑
Spiritual Support (e.g., chaplain, rabbi)	❑	❑	❑	❑
Others	❑	❑	❑	❑

How do the PPC units support the families, besides the provision of medical and nursing care?

	All units	Some units	No unit	I do not know
Case management	❑	❑	❑	❑
Social support	❑	❑	❑	❑
Legal support	❑	❑	❑	❑
Psychological counseling	❑	❑	❑	❑
Grief counseling	❑	❑	❑	❑
Psychotherapy for siblings/families	❑	❑	❑	❑
Spiritual support (e.g., chaplain, rabbi)	❑	❑	❑	❑
Other	❑	❑	❑	❑

Which patients are treated on the PPC units?

	On all units	On some units	On no unit	I do not know
Neonatal	❑	❑	❑	❑
Oncological patients, solid tumors	❑	❑	❑	❑
Oncological patients, leukemia	❑	❑	❑	❑
Non-oncological patients (not ventilated)	❑	❑	❑	❑
Non-oncological patients (ventilated)	❑	❑	❑	❑
Other	❑	❑	❑	❑

When may the services of the PPC units be used?

1. From the diagnosis of a life-limiting/life-threatening diagnosis onward, including the end-of-life period
2. Only during the end-of-life period
3. Other

How is the access to the PPC units regulated?

1. Access only via referral
2. Services may be privately arranged
3. Other

Do any age restrictions regarding the affected child exist for the utilization of the PPC units?

1. Yes
2. No

Is the utilization of PPC units limited in time? (e.g., 4 weeks per year)

1. Yes, utilization is limited in time
2. No, utilization is not limited in time

Is the utilization of PPC units free of charge for the families?

1. Yes
2. No

Do you have any further information on the PPC units in your country for us that we should consider when assessing the state of PPC in the EU?

Are Inpatient Children's and Adolescents' Hospices available in your country? Children's and Adolescents' Hospices are inpatient institutions that are managed by a professional. They provide respite care as well as terminal care to children and their families. For respite care, children may spend time in the hospice, with or without the family being present. For terminal care, children and their family, if desired, stay in the hospice until death.

1. Yes
2. No

Approximate number of pediatric hospices in your country: This information is very important for us. If you do not know the exact number of teams, please try to make an educated guess. Only in case you absolutely do not know, enter "000".

Approximate total number of beds of all pediatric hospices in your country (rough estimation ok)This information is very important for us. If you do not know the exact number of teams, please try to make an educated guess. Only in case you absolutely do not know, enter "000".

Where do pediatric hospices exist in your country?

1. Available in all regions
2. Available in most regions
3. Available only in a few regions
4. Other

How are the pediatric hospices mainly financed/funded?

1. Government funding
2. Health insurances
3. Donations
4. Other

Is there additional funding for the pediatric hospices?

1. Government funding
2. Health insurances
3. Donations
4. Other

Which professions are employed in the pediatric hospices?

	In all hospices	In some hospices	In no hospice	I do not know
Physicans	❑	❑	❑	❑
Nurses	❑	❑	❑	❑
Psychologists	❑	❑	❑	❑
Social workers	❑	❑	❑	❑
Volunteers	❑	❑	❑	❑
Spiritual Support (e.g., chaplain, rabbi)	❑	❑	❑	❑
Others	❑	❑	❑	❑

How do pediatric hospices support the families?

	All hospices	Some hospices	No hospice	I do not know
Medical Care	❑	❑	❑	❑
Nursing Care	❑	❑	❑	❑
Case management	❑	❑	❑	❑
Social support	❑	❑	❑	❑
Legal support	❑	❑	❑	❑
Psychological counseling	❑	❑	❑	❑
Psychotherapeutic support for siblings/families	❑	❑	❑	❑
Grief counseling	❑	❑	❑	❑
Bereavement support	❑	❑	❑	❑
Spiritual support (e.g. chaplain, rabbi)	❑	❑	❑	❑
Educational support	❑	❑	❑	❑
Cold room	❑	❑	❑	❑
Other	❑	❑	❑	❑

Which patients are accommodated in the pediatric hospices?

	On all hospices	On some hospices	On no hospice	I do not know
Neonatal	❑	❑	❑	❑
Oncological patients, solid tumors	❑	❑	❑	❑
Oncological patients, leukemia	❑	❑	❑	❑
Non-oncological patients (not ventilated)	❑	❑	❑	❑
Non-oncological patients (ventilated)	❑	❑	❑	❑
Other	❑	❑	❑	❑

When may the services of the pediatric hospices be used?

1. From the diagnosis of a life-limiting/life-threatening diagnosis onward, including the end-of-life period
2. Only during the end-of-life period
3. Other

How is the access to pediatric hospices regulated?

1. Access only via referral
2. Services may be privately arranged
3. Other

Do any age restrictions regarding the affected child exist for the utilization of pediatric hospices?

1. Yes
2. No

Is the utilization of pediatric hospices limited in time? (e.g., 4 weeks per year)

1. Yes, utilization is limited in time
2. No, utilization is not limited in time.

Is the utilization of pediatric hospices free of charge for the families?

1. Yes
2. No

How is the pediatric hospice operation regulated by the local/national health authorities?

Do you have any further information on pediatric hospices in your country for us that we should consider when assessing the state of PPC in the EU?

Are Outpatient children's hospice services (hospice-at-home) available in your country? Outpatient children's hospice services usually almost exclusively consist of volunteer workers who provide respite and psychosocial support. They support the family at home temporarily during the day and thereby allow for more free time in daily life.

1. Yes, available in my country
2. No, not available in my country

Approximate number of outpatient children's hospice services in your country: This information is very important for us. If you do not know the exact number of teams, please try to make an educated guess. Only in case you absolutely do not know, enter "000".

Where do outpatient children's hospice services exist in your country?

1. Available in all regions
2. Available in most regions
3. Available only in a few regions
4. Other

How are the outpatient children's hospice services mainly financed / funded?

1. Government funding
2. Health insurances
3. Donations
4. Other

Is there additional funding for the outpatient children's hospice services?

1. Government funding
2. Health insurances
3. Donations
4. Other

Who is part of the outpatient children's hospice services?

	For all teams	For some teams	For no team	I do not know
Volunteers	❑	❑	❑	❑
Professional manager	❑	❑	❑	❑
Other employees	❑	❑	❑	❑

How do the outpatient children's hospice services support the families?

	All teams	Some teams	No team	I do not know
Spending time with the affected child	❑	❑	❑	❑
Spending time with healthy siblings	❑	❑	❑	❑
Nursing support	❑	❑	❑	❑
Helping with household chores	❑	❑	❑	❑
Psychological support	❑	❑	❑	❑
Other	❑	❑	❑	❑

Do you have any further information on outpatient children's hospice services in your country for us that we should consider when assessing the state of PPC in the EU?

We have now asked you about: Specialized Pediatric Palliative Home Care (SPHHC), Specialized Clinical Consultation Services, Pediatric Palliative Care Units (PPC)Inpatient children's and adolescents' hospices, Outpatient children's hospice services. Are there other specialized pediatric palliative care services available in your country which we did not assess in this questionnaire?

1. Yes, there are.
2. No, there are not.

How many years of work experience do you have within PPC?

What is your profession?

1. Physician
2. Nurse
3. Psychologist
4. Social worker
5. Other

How are you involved in PPC?

1. Actively providing care
2. Administrative services
3. Active membership in PPC networks/associations
4. Passive membership in PPC networks/associations
5. Other

Would you like to become a member of the European Network for Pediatric Palliative Care that we aim to establish with the collected data?

1. Yes, please keep me updated.
2. No, thank you.

This is the end of the questionnaire. Thank you very much for providing us with data on specialized pediatric palliative care in your country!
