# Supplementary material for: Expert survey on coverage and characteristics of pediatric palliative care in Europe – a focus on home care
Source: BMC Palliat Care. 2022 Oct 17;21:185. doi: 10.1186/s12904-022-01078-0 (PMC9575204; doi:10.1186/s12904-022-01078-0)
Supplement: Supplementary file 2 — Additional file 2: Supplemental Material 2. Topic blocks and definitions within the utilized pediatric palliative care questionnaire. [file 12904_2022_1078_MOESM2_ESM.pdf]

Supplemental Material 2. Topic blocks and definitions within the utilized pediatric palliative care questionnaire.

Topic	Definition
**Pediatric palliative care services**	
Pediatric palliative home care	The main objective of pediatric palliative home care teams is to provide and/or coordinate palliative care for children (0-18 years old) with life-threatening or life-limiting conditions at home. The team is managed by a professional and furthermore almost exclusively consists of professionals, though it may be supported by volunteers.
Clinical consultation services	Clinical consultation services work inside the hospital and support families and other physicians/nurses when children and their families have palliative care needs, irrespective of their diagnosis.
Pediatric palliative care unit	A Pediatric palliative care unit is a hospital unit that exclusively admits pediatric palliative care patients. Pediatric palliative care units do not provide respite care. Pediatric beds on palliative care units for adults are not considered a pediatric palliative care unit.
Inpatient children's and adolescents' hospices	Inpatient children's and adolescents' hospices are inpatient institutions that are managed by a professional. They provide respite care as well as terminal care to children and their families. For respite care, children may spend time in the hospice, with or without the family being present. For terminal care, children and their family, if desired, stay in the hospice until death.
Outpatient children's hospice service (hospice-at-home)	Outpatient children's hospice services usually almost exclusively consist of volunteer workers who provide respite and psychosocial support. They support the family at home temporarily during the day and thereby allow for more free time in daily life.
**National pediatric palliative care activities**	
Pediatric palliative care plan/strategy	(No definition provided)
Pediatric palliative care networks, expert associations or taskforces	A national network of PPC is formed by people interested in the advancement of PPC provision in a specific country and must be a registered association.
Pediatric palliative care documents	Official documents specify requirements that PPC services should adhere to. They also demonstrate guidelines for the development, equipment and configuration of services required for adequate structural quality.
