# Supplementary material for: Expert survey on coverage and characteristics of pediatric palliative care in Europe – a focus on home care
Source: BMC Palliat Care. 2022 Oct 17;21:185. doi: 10.1186/s12904-022-01078-0 (PMC9575204; doi:10.1186/s12904-022-01078-0)
Supplement: Supplementary file 3 — Additional file 3: Supplemental Material 3. Specialized pediatric palliative home care team composition. [file 12904_2022_1078_MOESM3_ESM.pdf]

Supplemental Material 3. Specialized pediatric palliative home care team composition.

physicians	nurses	psychologists	social worker	volunteers	grief counselor	spiritual support	others	*n* (%)
no multi-professionalism								
								1 (25)
■								*1* (25)
	■							*2* (50)
								4 (100)
multi-professionalism								
■	■							*5* (29.4)
■	■	■	■					*3* (17.6)
■	■	■	■	■		■		*2* (11.8)
■	■	■	■	■	■	■		*1* (5.3)
■	■	■	■			■	■	1 (5.3)
■	■	■	■	■	■			*1* (5.3)
■	■		■		■	■	■	*1* (5.3)
■	■	■	■			■		*1* (5.3)
■	■	■		■				*1* (5.3)
■	■		■				■	*1* (5.3)
								17 (100)

*Only those 21 countries are listed where the corresponding professions are available in all national specialized pediatric palliative home care teams.
